# The Effect of Electrochemical Composite Coatings with LaF_3_-LaB_6_ Particles in Nickel–Copper Matrix on the Metallurgical Processes in Arc Welding of Low Alloy Ferrite-Pearlite Steels

**DOI:** 10.3390/ma14061509

**Published:** 2021-03-19

**Authors:** Sergey G. Parshin, Victor A. Karkhin, Peter Mayr, Alexey S. Maystro

**Affiliations:** 1 Institute of Mechanical Engineering, Materials and Transport, Peter the Great St.Petersburg Polytechnic University, Polytechnicheskaya, 29, St. Petersburg 195251, Russia; victor.karkhin@mail.ru (V.A.K.); maystro@spbstu.ru (A.S.M.); 2 Chair of Materials Engineering of Additive Manufacturing, Department of Mechanical Engineering, Technical University of Munich, Boltzmann str. 15, 85748 München, Germany; peter.mayr@tum.de

**Keywords:** welding metallurgy, welding consumables, rare earth compounds, electrochemistry, composite coatings, low alloy steel, microstructural refinement

## Abstract

Development of welding consumables with fluorides and borides of rare earth metals is a promising area for improving the weldability of low alloy steels. As lanthanum fluoride and boride dissociate, lanthanum and boron dissolve in the weld pool and the welding arc plasma is saturated with fluorine. As a result of FeO, MnO, SiO_2_ deoxidation and FeS, MnS desulfurization, refractory lanthanum sulfides and oxides La_2_O_3_, La_2_S_3_ are formed in the weld pool, which can be the crystallization nuclei in the weld pool and the origin of acicular ferrite nucleation. The paper proposes a model of metallurgical processes in the arc and weld pool, as well as a model of electrochemical adsorption of Ni^2+^ cations in colloidal electrolytes during electrostatic deposition of nano-dispersed insoluble particles of LaF_3_ or LaB_6_ on the surface of wire. The paper discusses the constructional design of the welding wire and the technology for forming electrochemical composite coatings with copper and nickel matrix. The composite wires applied in the welding of low alloy steels make it possible to refine the microstructure, increase the tensile strength by 4% and the impact toughness of welds by 20%.

## 1. Introduction

Low alloy steels are widely used to produce welded structures in the ship-building, oil and gas pipeline transportation, wind turbines, and offshore platforms. Welded steel structures are used at low temperatures, often in hydrogen-containing and aggressive environments. The weldability of steels becomes more complicated due to the grain coarsening, hydrogen embrittlement, hydrogen-assisted cracking (HAC), and reduced impact toughness at low temperatures [1,2]. The main method that is used to weld these steels is a metal active gas (MAG) and metal inert gas (MIG) welding with solid wire, because it ensures the lowest level of diffusible hydrogen in welds [3,4].

The microstructural state of the weld metal and the heat-affected zone is the most important factor in the mechanism of hydrogen embrittlement, cracking and reduction in impact toughness [5,6]. The desired fine-grained microstructure is often achieved in welding using an optimal thermal cycle [7,8,9] and through minimizing the content of impurities and segregations making the grain structure more refined [10]. Another effective metallurgical solution is to refine the microstructure using a mechanism of heterogeneous grain nucleation at non-metallic inclusions and acicular ferrite when refractory oxides, sulfides, and nitrides are formed [11]. The most effective method for refining the microstructure is to design special welding wires and introduce rare earth metals (REMs) [12].

The refractory REM oxides and sulfides of type REM_2_O_3_, REM_2_S_3_ are non-metallic inclusions having a spherical shape similar to titanium oxides TiO, TiO_2_, Ti_2_O_3_ [13,14]. The oxides and sulfides of REMs have high melting temperatures, °C: La_2_O_3_ (2304); Y_2_O_3_ (2439); Ce_2_O_3_ (2250); ThO_2_ (3350); La_2_S_3_ (2110); Y_2_S_3_ (1925); Ce_2_S_3_ (2450); Th_2_S_3_ (2300) [15]. Thus, all these compounds formed in the weld pool increase the amount of crystallization nuclei, which contributes to the grain refinement of the microstructure and acicular ferrite nucleation [16,17]. Non-metallic spherical inclusions TiO, TiO_2_, Ti_2_O_3_ can also be the effective nuclei of acicular ferrite formation [18,19,20,21,22]. However, REMs have an advantage because they are more active in desulfurization and deoxidation reactions [23,24].

It is essential to highlight the importance of the borides of REMs in the mechanism of strengthening and refinement of the microstructure through the formation of refractory boron carbides B_4_C and boron nitrides BN [25]. Introducing boron has a positive effect on the properties and microstructure of cold-resistant austenitic steel with a small residual content of nitrogen being below 0.04% and boron being below 0.004%. This effect is related to the solubility of boron in γ-Fe and α-Fe, the reduction in harmful impurities of sulfur S and phosphorus P, and the formation of borides Me_2_B, Me_5_B_3_, Me_3_(C,B), Me_23_(C,B)_6_, FeMo_2_B_4_ [26].

In welding metallurgy, an important role is the formation of free fluorine as fluorides dissociate. It reduces the content of diffusible hydrogen due to the reactions in which hydrogen fluoride HF is formed [27]. The high effectiveness of fluorine and fluorides in removing hydrogen is explained by the metallurgical activity of fluorine and fluorides in the reactions with atomic H, molecular H_2_, and water vapor H_2_O both in liquid slag and in arc welding plasma [28].

Since pure metallic powders of REMs and boron have a high chemical activity, their direct introduction during welding leads to losses due to oxidation and evaporation. That is why to increase the transition coefficient of micro-alloying elements of REMs and boron in the weld pool, high-melting point compounds of REMs can be introduced in the form of fluorides and borides, whose properties are shown in Table 1.

The research is aimed at improving the mechanical properties of welds, refinement of the microstructure of welds using newly-designed welding wires with electrochemical composite coatings that contain lanthanum fluoride and boride nano-dispersed particles for welding low alloy ferrite-pearlite steels.

## 2. Materials and Methods

The arc welding was focused on using plates of S355 steel according to EN10025-3:2019 (10HSND steel according to GOST 19281-2014 in Russian) of 300 mm × 150 mm × 14 mm with G3Si1 wire (ESAB, Gothenburg, Kingdom of Sweden) according to EN ISO 14341-A, 1.2 mm in diameter, plates of S355 steel according to EN10025-3:2019 (09G2S steel according to GOST 19281-2014 in Russian) of 400 mm × 150 mm × 16 mm with S2Mo wire (ESAB, Gothenburg, Kingdom of Sweden) according to EN ISO 14171-A, 3 mm in diameter. In order to make composite wires, standard G3Si1, S2Mo wires were used with their surface being coated in composite electrochemical layers, 5–15 µm in thickness. The coatings consisted of a copper and nickel matrix and nano-dispersed LaF_3_ and LaB_6_ particles with a diameter of 0.3–0.7 µm. The parameters of the electrochemical process are shown in Table 2.

**Figure 1:**
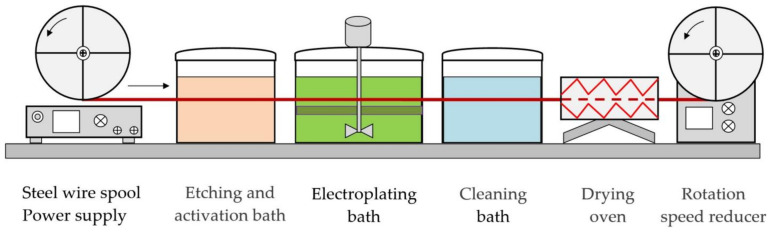
Schematic of the set-up used for treating welding wire and forming composite coatings.

The set-up shown in Figure 1 was used to deposit composite coatings on welding wire.

In the technology that is used to form composite coatings, the welding wire goes in the continuous process into the etching and surface activation bath from 10% aqueous solution of H_2_SO_4_ at a feed rate of 0.5–2.5 m/min. The treatment at a temperature of 25 °C removes impurities, organic contaminants, and oxide films from the wire surface. Then the wire goes into the main electroplating bath containing colloidal electrolyte with nano-dispersed LaF_3_, LaB_6_ particles where the solution is thoroughly stirred. The processing time of the wire in each bath is 7–36 s. After the composite coating is formed, the wire goes into another bath of distilled water at a temperature of 60 °C to be cleansed from the remains of the electrolyte and then into the drying oven at a temperature of 115 °C. After that, the wire is wound in a coil.

**Figure 2:**
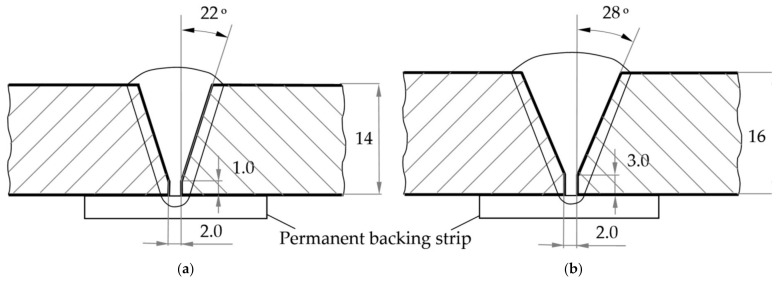
The design of butt joints: (**a**) in metal active gas (MAG) welding according to GOST 14771-76 in Russian; (**b**) in submerged arc welding (SAW) process according to GOST 8713-79 in Russian.

**Figure 3:**
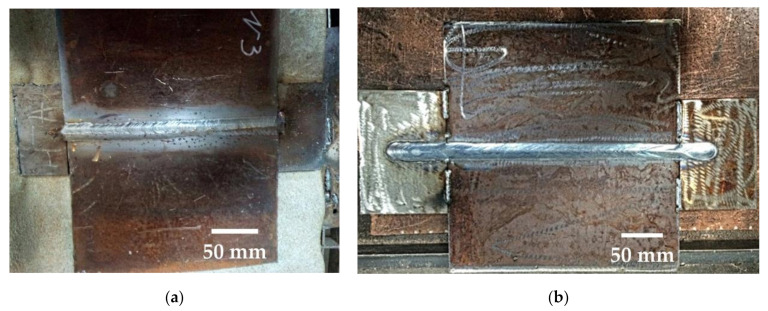
The appearance of welds: (**a**) from 10HSND steel; (**b**) from 09G2S steel.

For MAG-welding of samples using a mixture of 82% of Ar and 18% of CO_2_, the following power sources were used an ESAB Origo MIG L405. For submerged arc welding (SAW) of plates made of 09G2S steel under a layer of OK Flux 10.71 (SiO_2_ + TiO_2_—20%; CaO + MgO—25%; Al_2_O_3_ + MnO—35%; CaF_2_—15%), an A2 Multitrack automatic welding machine with an LAF 1001 power source was used. The welding parameters are given in Table 3. The design of joints and appearance of the welds is shown in Figure 2 and Figure 3.

The metallurgical reactions and phase compositions were calculated using thermodynamic simulations based on the data of individual substances and using the software called IVTANTHERMO (Joint Institute for High Temperatures of the Russian Academy of Sciences, Moscow, Russia) and FactSage (CRCT, Montreal, Canada) [29,30]. A Tinius Olsen Model 602 machine (Tinius Olsen, Horsham, PA, USA) according to ISO 4136:2012, a PH450 pendulum impact testing machine (Walter + Bai AG, Löhningen, Switzerland) according to the standard testing of Charpy V-notch specimens to ISO 9016:2012, and an EMCOTEST DuraScan-20 hardness tester (EMCO-TEST PrufmaSchinen GmbH, Kuchl, Austria) according to ISO 9015-1:2001 were used for mechanical testing. The chemical composition was assessed using a Bruker Q4 TASMAN optical emission spectrometer (Bruker, Karlsruhe, Germany). Reichert-Jung Me F3A, Zeiss Axiovert 200 MAT microscopes (Carl Zeiss AG, Oberkochen, Germany) were used for optical metallography, while a SEM TESCAN MIRA 3 scanning microscope (Tescan Orsay Holding, Brno, Czech Republic) and a Bruker D8 Advance diffractometer (Bruker, Karlsruhe, Germany) were applied for X-ray analysis.

## 3. Results and Discussion

**Figure 4:**
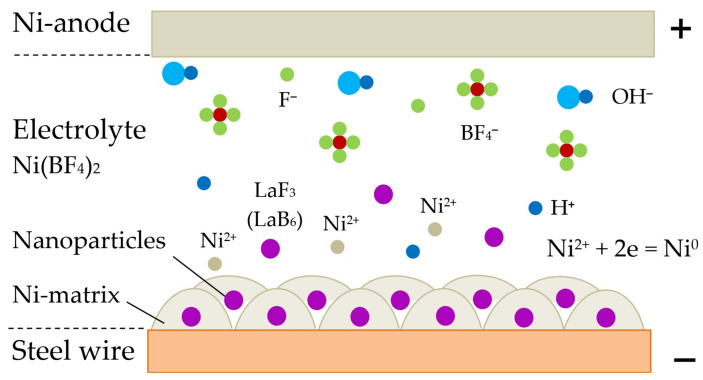
The mechanism of electrochemical ion adsorption and formation of the composite coating in the colloidal solution of Ni(BF_4_)_2_ in ethanol C_2_H_6_O containing nano-dispersed insoluble LaF_3_ or LaB_6_ particles.

The mechanism according to which the composite coatings are formed in electrolyte with particles of rare earth compounds is shown in Figure 4.

Affected by electrostatic forces, insoluble LaF_3_ or LaB_6_ nanoparticles are attracted and deposited on the surface of the wire having a negative charge. The nickel cations Ni^2+^ or copper cations Cu^2+^ from the electrolyte and from the anode are neutralized on the surface of the cathode and form a metal layer over nanoparticles. OH^−^, F^−^, BF^4−^ anions, which move in the opposite direction, are neutralized on the surface of the nickel anode and contribute to its erosion and enrichment of the electrolyte by nickel cations Ni^2+^. Thus, in a fast process, the composite is formed from the metallic matrix and nanoparticles. In case the composite Cu-coating is formed in electrolyte, copper cations Cu^2+^ and anions SO^4−^ appear from copper sulfate CuSO_4_, and pure copper is used as anode.

**Figure 5:**
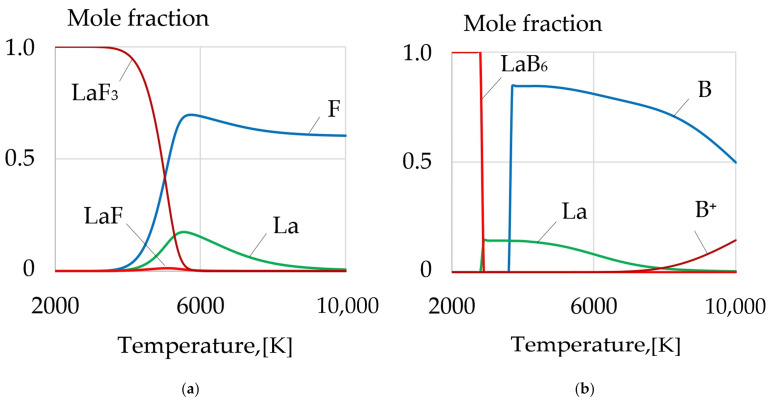
The mole fraction of components during dissociation: (**a**) LaF_3_; (**b**) LaB_6_.

During welding, the wire with a composite coating consisting of nanoparticles and lanthanum compounds LaF_3_, LaB_6_ is melted and the molecules in the welding arc evaporate and dissociate into atomic La, F, B, as shown in Figure 5. 

Then the atoms are ionized, which is proven by thermodynamic simulation of the phase composition, as shown in Figure 5. The dissociation products LaF_3_, LaB_6_ in the form of rare earth elements and boron are adsorbed on the melted surface of the wire and on the surface of the weld pool, after which they dissolve in the liquid metal. This helps to increase the activity of metallurgical processes by removing detrimental compounds and gases from the weld pool.

**Figure 6:**
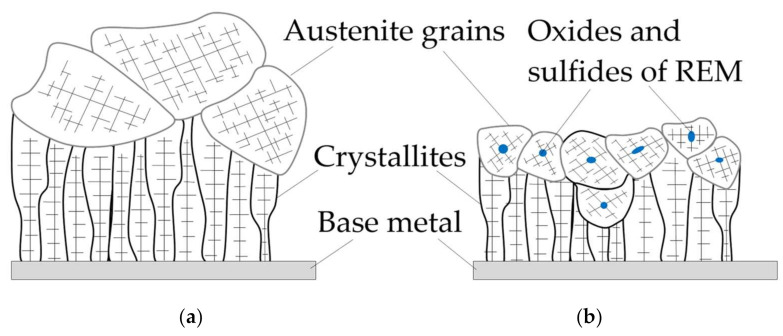
The model of dendritic crystallization at the boundary of “weld pool-base metal” transition: (**a**) without limiting the growth of crystallites; (**b**) with limiting the growth of crystallites involving additional crystallization nuclei, oxides, and sulfides.

The weld pool always contains detrimental oxides FeO, SiO_2_, MnO, sulfides FeS, MnS, as well as diffusible elements S, H, N, which reduce the impact toughness, plasticity of welds and increase their sensitivity to hydrogen-assisted cracking. Enriching the weld pool with rare earth elements and boron leads to the interaction with harmful compounds and, as a result, refractory oxides and sulfides of REMs as well as boron nitrides BN are formed. They are the primary crystallization nuclei. Increasing the amount of refractory crystallization nuclei prevents large crystallites and dendrites from growing, which contributes to the refining of the primary austenite grains, as shown in Figure 6.

**Figure 7:**
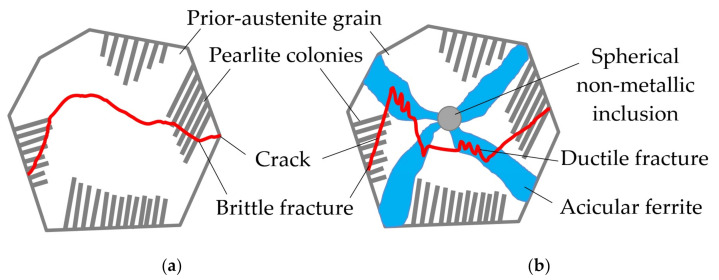
Cold crack propagation trajectory in prior-austenite grains: (**a**) without acicular ferrite; (**b**) with acicular ferrite nucleated on a spherical non-metallic inclusion.

A similar mechanism of dendritic crystallization with additional crystallization nuclei, oxides, and sulfides is shown in other publications [31,32,33,34,35]. The austenitic grain size in the dendritic crystallization is reduced from 770 to 344 µm in columnar and equiaxed zones of primary crystallites, as shown in the study by Salas-Reyes et al. [36]. In case of further cooling, secondary crystallization takes place in pearlitic and bainitic transformation, and intragranular acicular ferrite nucleates inside austenite grains on the surface of refractory non-metallic inclusions, as shown in Figure 7. 

This restrains cold cracks from propagation and increases the length its trajectory. Thus, oxides and sulfides of rare earth metals and boron nitride can stimulate the refining of the primary austenite microstructure and acicular ferrite nucleation in ferrite-pearlite steels [37,38,39]. Increasing the volume fraction of acicular ferrite and refining the microstructure of welds is an essential condition for increasing the impact toughness, reducing the critical temperature of ductile-brittle transition and decreasing the sensitivity to hydrogen-assisted cracking [40].

**Figure 8:**
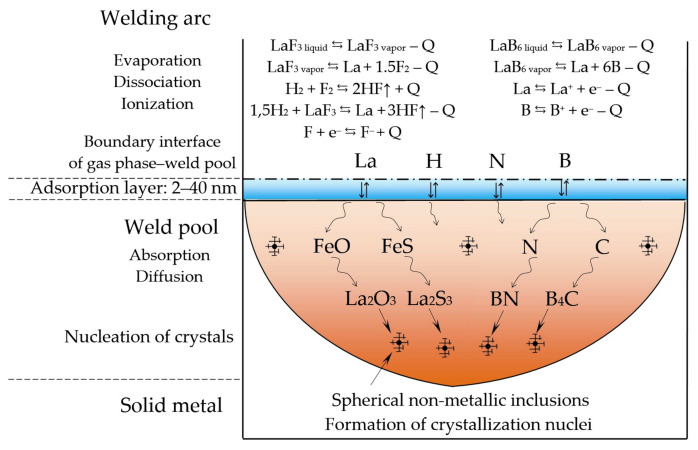
The model of metallurgical processes occurring in the welding arc and in weld pool when lanthanum fluoride and lanthanum boride are introduced and refractory non-metallic inclusions are formed.

The model of metallurgical processes occurring in the welding arc and weld pool as LaF_3_ and LaB_6_ are introduced is shown in Figure 8.

After LaF_3_ and LaB_6_ dissociate, micro-alloying elements La and B are adsorbed on the surface of the weld pool and then dissolve in the weld pool. The thickness of the adsorption layer depends on the surface energy and gas composition. The adsorption layer thickness can be determined by the calculation method according to the Gibbs–Guggenheim definition [41]. According to the data by Lapshin et al. [42] the thickness of the adsorption layer reaches up to 40 nm. When the elements are distributed in the melt, there is metallurgical reaction of La with oxide FeO and sulfide FeS, and refractory oxide La_2_O_3_ and sulfide La_2_S_3_ are formed. When boron interacts with dissolved nitrogen and carbon, nitride BN and carbide B_4_C are formed. Apart from these reactions, possible reactions may include lanthanum and boron interacting with oxides and sulfides MnO, MnS, SiO_2_, as well as with diffusible elements—absorbed hydrogen, nitrogen, dissolved sulfur, and free oxygen. Since the atmosphere of the arc often contains water vapor and hydrogen, the binding of molecular hydrogen H_2_ in the atmosphere of the arc is important in compounds HF during the direct reaction of H_2_ interacting with fluorides of REMs and free fluorine.

A detailed analysis of the reactions between REMs and boron with diffusible elements as well as interaction with hydrogen in the welding arc plasma needs to be described separately and is outside the scope of this research.

**Figure 9:**
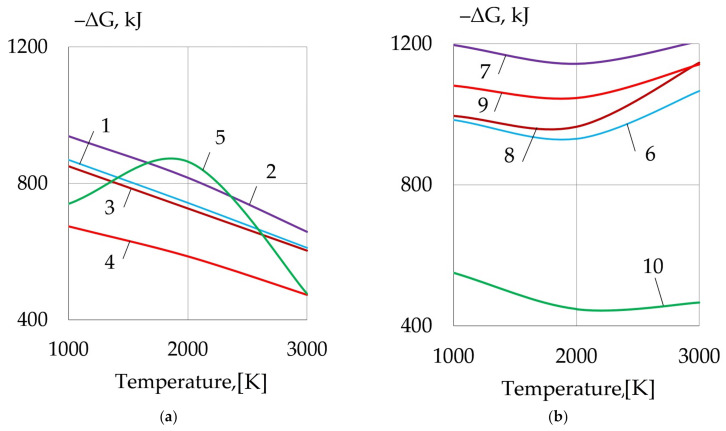
Change in Gibbs free energy of the reactions: (**a**) iron deoxidation and desulfurization (1–5); (**b**) silicon deoxidation and manganese desulfurization (6–10).

As a result of the metallurgical interaction, refractory non-metallic inclusions are formed in the weld pool, which are crystallization nuclei. The formation of the refractory oxides, sulfides and boron nitride can be explained with the high activity of dissolved rare earth elements and boron in the deoxidation and desulfurization reactions. The thermodynamic simulation confirms that the metallurgical reactions of deoxidation of FeO, SiO_2_ and desulfurization of FeS, MnS are highly probable. As rare earth metals La, Ce, Y, Th and boron are adsorbed and then dissolve in the weld pool, refractory oxides and sulfides of REMs, and boron nitride BN are formed according to Reactions (1)–(10), which have negative values of Gibbs free energy, as shown in Figure 9.

1.5FeO + 1.5FeS + 2La = 0.5La_2_O_3_ + 0.5La_2_S_3_ + 3Fe (1)

1.5FeO + 1.5FeS + 2Y = 0.5Y_2_O_3_ + 0.5Y_2_S_3_ + 3Fe (2)

1.5FeO + 1.5FeS + 2Ce = 0.5Ce_2_O_3_ + 0.5Ce_2_S_3_ + 3Fe (3)

FeO + 1.5FeS + 1.5Th = 0.5ThO_2_ + 0.5Th_2_S_3_ + 2.5Fe (4)

1.5FeO + 1.5FeS + N + 3B = 0.5B_2_O_3_ + 0.5B_2_S_3_ + BN + 3Fe (5)

3MnS + 1.5SiO_2_ + 4La = La_2_O_3_ + La_2_S_3_ + 3Mn + 1.5Si (6)

3MnS + 1.5SiO_2_ + 4Y = Y_2_O_3_ + Y_2_S_3_ + 3Mn + 1.5Si (7)

3MnS + 1.5SiO_2_ + 4Ce = Ce_2_O_3_ + La_2_S_3_ + 3Mn + 1.5Si (8)

3MnS + SiO_2_ + 3Th = ThO_2_ + Th_2_S_3_ + 3Mn + Si (9)

MnO + SiO_2_ + N + 3B = B_2_O_3_ + BN + Mn + Si (10)

**Figure 10:**
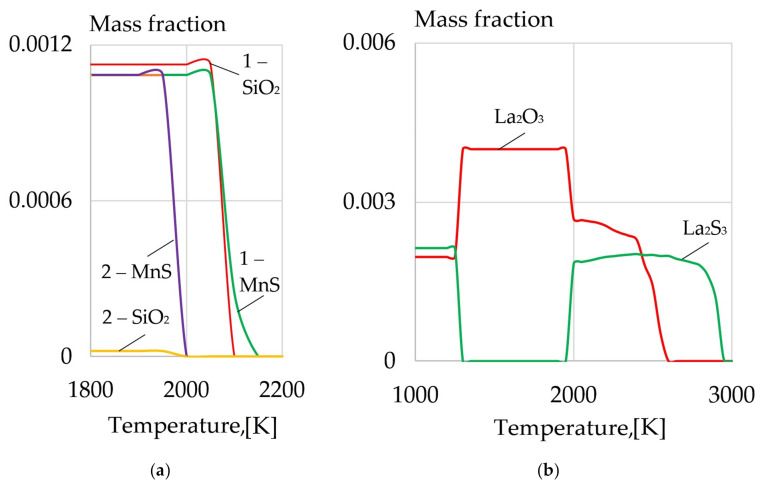
(**a**) The content of SiO_2_ and MnS in 10HSND steel: (1) without REMs, (2) with addition of 1% mixture of LaF_3_ and LaB_6_; (**b**) the content of La_2_O_3_ and La_2_S_3_ in 10HSND steel.

**Figure 11:**
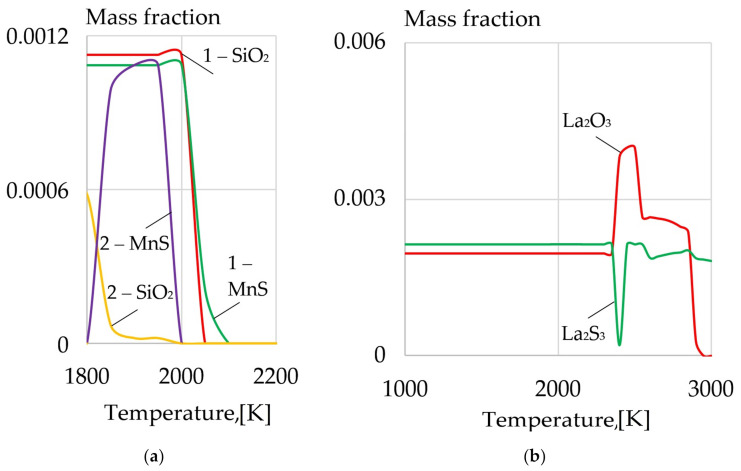
(**a**) The content of SiO_2_ and MnS in 09G2S steel: (1) without REMs, (2) with addition of 1% mixture of LaF_3_ and LaB_6_; (**b**) the content of La_2_O_3_ and La_2_S_3_ in 09G2S steel.

The thermodynamic modeling of the phase composition of the weld pool as 10HSND and 09G2S steels melt proves that with the introduction of LaF_3_ and LaB_6,_ refractory sulfides and oxides of REMs are formed, as shown in Figure 10 and Figure 11. It causes the reduction in the content of S, O, SiO_2_, which are harmful impurities embrittling the weld because of the low-melting eutectics Fe-FeO, Fe-FeS, formed at the grain boundaries.

**Figure 12:**
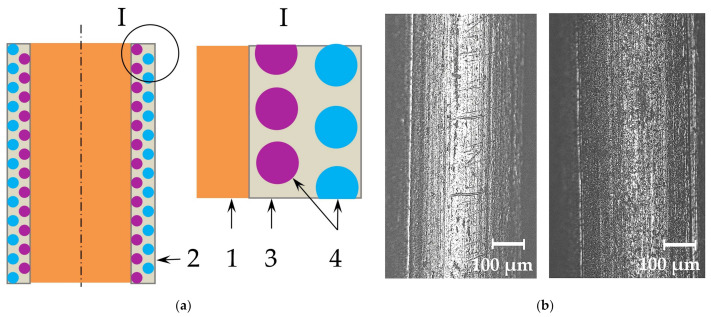
(**a**) The design of the composite wire: (1) solid wire, (2) composite coating, (3) metal matrix, (4) particles of rare earth compounds, (**b**) optical macrostructure of the surface of G3Si1 wire with Ni-LaF_3_ coating; (**c**) optical macrostructure of the surface of G3Si1 wire with Ni-LaB_6_ coating.

For effective introduction of REMs in the weld pool during welding of pearlitic steels, special design of the welding wire has been worked out. The design and the macrostructure of the composite wire are shown in Figure 12.

The wire is covered in an electrochemical composite coating with particles of REMs, which are evenly distributed inside the metal matrix coating. It ensures good electrical conductivity of the surface of the wire, which is important in the processes of automated and robotic welding.

**Figure 13:**
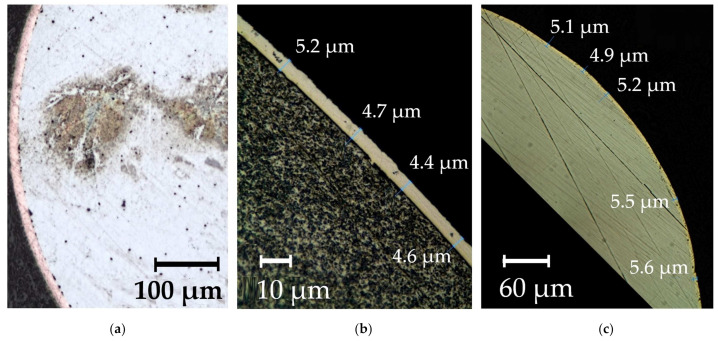
The optical microstructure of composite coatings on G3Si1 wire, 1.2 mm in diameter: (**a**) Cu-LaF_3_ coating; (**b**) Ni-LaF_3_ coating; (**c**) Ni-LaB_6_ coating.

The electrochemical technology that is used to form nanocomposite coatings ensures high density and adhesion of the composite coating to the steel wire surface. Figure 13 shows the microstructure of the composite coatings on G3Si1 wires.

**Figure 14:**
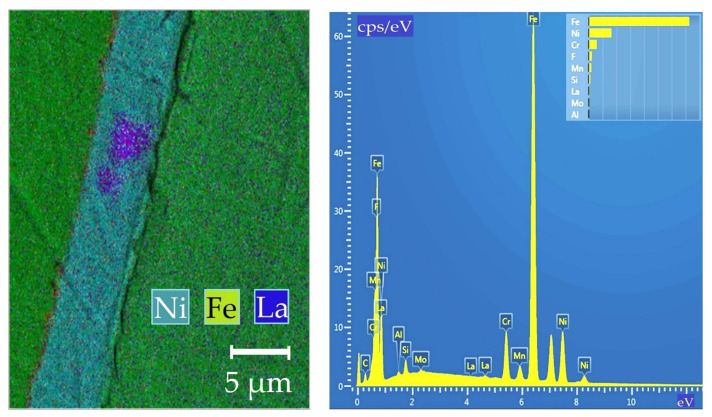
SEM-elemental mapping of Ni-LaF_3_ composite coating.

Figure 14 shows a typical SEM-elemental mapping of the composite Ni-LaF_3_ coating on G3Si1 wire.

According to X-ray structural analysis, typical Ni-LaF_3_ coating being about 5 µm in thickness contains up to 1.6 wt.% of La; up to 2 wt.% of La_2_Ni_3_; up to 0.2 wt.% of NiF_3_; and up to 96 wt.% of Ni.

The chemical analysis of weld metal in the weld center in 10HSND steel being 14 mm in thickness showed that as composite wires are applied, Cu, Ni, La, and Mn content insignificantly is increased. The content of other chemical elements did not change, as shown in Table 4.

According to the investigation of the mechanical properties of welds of 10HSND steel, using wire with Cu-LaF_3_ composite coating leads to an increase in tensile strength by 4%, and in impact toughness by 9.6–13.9%, as shown in Table 5.

Studying the mechanical characteristics of the welds on the plates made of 09G2S steel during submerged arc welding shows that if the wire with Ni-LaF_3_ composite coating is used, the impact toughness increases in the weld by 20.3%, and in the heat-affected zone by 11.4%, as shown in Table 6.

**Figure 15:**
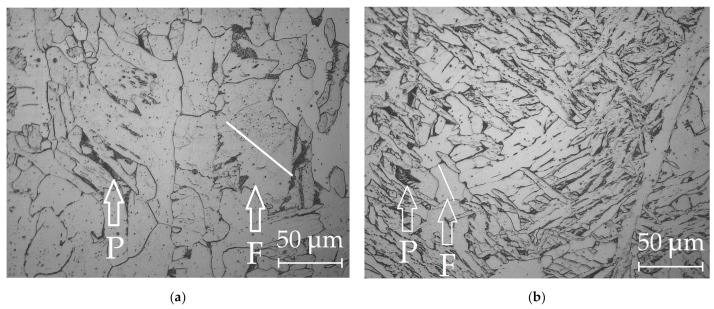
The typical microstructure of the in the weld metal center: (**a**) standard G3Si1 wire; (**b**) G3Si1 wire with Ni-LaF_3_ coating. The arrows indicate the grains of ferrite (F) and pearlite (P).

The improved mechanical properties are due to the refinement of the microstructure, as shown in Figure 15.

The improvement of the microstructure is a result of the positive effect of rare earth compounds in the reactions of deoxidation and refining of grain boundaries from harmful segregations and impurities. Another reason for the improvement of mechanical properties can be the grain refinement due to the formation of refractory oxides and sulfides of lanthanum and the inhibition of growth of primary crystallites during solidification. The analysis of the microstructure of the weld metal shows that using composite wires with nano-dispersed particles of LaF_3_ and LaB_6_ leads to refinement of the microstructure, to a reduced average size of the grain from 40–60 µm to 12–28 µm for G3Si1 wire, to better morphology and even distribution of microstructural phases.

## 4. Conclusions

1. A model of the metallurgical processes for arc welding of low alloy pearlitic steels was proposed. It considers the formation of refractory sulfides and oxides of REMs as the crystallization nuclei in the weld pool and centers of acicular ferrite nucleation. Refractory non-metallic inclusions of oxides and sulfides of type REM_2_O_3_, REM_2_S_3_, and nitride BN can be formed in the weld pool due to a mechanism of the exchange reactions of FeO, MnO, SiO_2_ deoxidation and FeS, MnS desulfurization as they interact with rare earth elements La, Y, Ce, Th, and boron B. During the heating of the composite coating, the fluorides and borides of REMs dissociate and the micro-alloying elements are adsorbed on the surface of the molten droplets and weld pool. As a result, the weld pool is saturated with REMs and boron and active metallurgical treatment becomes possible. Due to the formation refractory sulfides and oxides of REMs, the growth of crystallites and austenite grains is limited, the microstructure is refined, and the strength and impact toughness of the welds at low temperatures increases.
2. Improving weldability and microstructure of pearlitic steel welds can be achieved thanks to the progressive design of the composite wire and the technology of its manufacturing, which imply that the composite coating is formed from copper and nickel matrix with nano-dispersed particles of fluorides and borides of REMs. A model is suggested for electrochemical adsorption of Ni^2+^ and Cu^2+^ cations with electrostatic deposition of nano-dispersed insoluble particles on the wire surface and subsequent formation of composite coatings in colloidal electrolytes based on Ni(BF_4_)_2_ and CuSO_4_ in ethanol C_2_H_6_O and distilled water H_2_O.

## Figures and Tables

**Table 1:** Physical properties of fluorides and borides of rare earth metals (REMs) [15].

REM	Melting Temperature, °C	Density, g/cm^3^	REM	Melting Temperature, °C	Density, g/cm^3^
LaF_3_	1493	5.9	LaB_6_	2715	4.76
CeF_3_	1430	6.157	CeB_6_	2550	4.87
YF_3_	1155	4.01	YB_6_	2600	3.72
NdF_3_	1377	6.51	NdB_6_	2610	4.93
ThF_4_	1110	6.1	ThB_6_	2450	6.99

**Table 2:** Parameters of the electrochemical process and electrolyte composition.

Mass Concentration, kg/m^3^	Temperature, °C	Electrolyte pH	Current Density, A/dm^2^	Current, A	Voltage, V	Wire Feed Rate, m/min
Ni(BF_4_)_2_·6H_2_O—500; NiCl_2_—70; LaF_3_—50 or LaB_6_—50; Ethanol·C_2_H_6_O—solvent	60–70	0.5–1	20–50	0.9–1.5	8–12	1.0–2.5
CuSO_4_·5H_2_O—250; H_2_SO_4_—70; LaF_3_—50 or LaB_6_—50; Distilled water H_2_O—solvent	25–30	1–1.5	5–8	1–2	10–14	0.5–1.5

**Table 3:** Welding parameters for 10HSND, 09G2S.

Weld Passes	10HSND with G3Si1 1.2 mm Mechanized Welding			09G2S with S2Mo 3.0 mm Automatic Welding		
	Current, A	Voltage, V	Wire Feed Rate, m/min	Current, A	Voltage, V	Travel Speed, m/min
**First Root Pass**	135	19	3.6	–	–	–
**Filling Passes**	215	23	5.8	420–430	33	0.35

**Table 4:** Chemical composition of the welds of 10HSND steel in the weld center with G3Si1 wire, wt.%.

**Base Metal**	**C**	**Si**	**Mn**	**Cr**	**Mo**	**Ni**	**Al**
	0.08–0.09	0.95–0.98	0.55–0.56	0.82–0.84	–	0.53–0.55	–
	**V**	**Cu**	**Ti**	**La**	**B**	**P**	**S**
	–	0.45–0.47	–	–	–	<0.006	<0.006
**Weld Metal** **G3Si1 Wire**	**C**	**Si**	**Mn**	**Cr**	**Mo**	**Ni**	**Al**
	0.05–0.06	0.79–0.86	1.07–1.08	0.14–0.15	–	0.09–0.11	–
	**V**	**Cu**	**Ti**	**La**	**B**	**P**	**S**
	–	0.09–0.1	–	–	–	<0.01	<0.008
**Weld Metal** **G3Si1 Wire with Cu-LaF_3_ Coating**	**C**	**Si**	**Mn**	**Cr**	**Mo**	**Ni**	**Al**
	0.09–0.1	0.8–0.95	1.13–1.16	0.2–0.23	–	0.12–0.17	–
	**V**	**Cu**	**Ti**	**La**	**B**	**P**	**S**
	–	1.2–1.7	–	0.003–0.004	–	<0.01	<0.006
**Weld Metal** **G3Si1 Wire with Ni-LaF_3_ Coating**	**C**	**Si**	**Mn**	**Cr**	**Mo**	**Ni**	**Al**
	0.06–0.07	0.81–0.85	1.09–1.11	0.1–0.12	–	0.25–0.28	–
	**V**	**Cu**	**Ti**	**La**	**B**	**P**	**S**
	–	0.08–0.10	–	0.002–0.004	–	<0.01	<0.008
**Weld Metal** **G3Si1 Wire with Ni-LaB_6_ Coating**	**C**	**Si**	**Mn**	**Cr**	**Mo**	**Ni**	**Al**
	0.05–0.06	0.83–0.86	1.14–1.15	0.09–0.1	–	0.3–0.32	–
	**V**	**Cu**	**Ti**	**La**	**B**	**P**	**S**
	–	0.07–0.08	–	0.001–0.003	<0.001	<0.01	<0.007

**Table 5:** The mechanical properties of the welds of 10HSND steel with G3Si1 wire.

Wire	Yield Strength, MPa	Tensile Strength, MPa	Elongation, %	Impact Toughness, KCV_+20_, J	Hardness, HV_10_	
				Weld Metal	Weld Metal	HAZ
G3Si1 Wire	420–430 425	575–580 577.5	22–24 23	186–188 187	186–208 197	203–236 219.5
G3Si1 Wire with Cu-LaF_3_ Coating	420–430 425	580–590 585	23–24 23.5	208–218 213	203–216 209.5	239–256 247.5
G3Si1 Wire with Ni-LaF_3_ Coating	425–435 430	597–602 599.5	23–25 24	183–211 197	199–210 204.5	196–228 212
G3Si1 Wire with Ni-LaB6 Coating	415–425 420	600–602 601	24–26 25	192–218 205	222–232 227	217–229 223

**Table 6:** The mechanical properties of the welds of 09G2S steel with S2Mo wire.

Wire	Yield Strength, MPa	Tensile Strength, MPa	Elongation, %	Impact Toughness, KCV_−40_, J		Hardness, HV_10_	
				Weld Metal	HAZ	Weld Metal	HAZ
S2Mo Wire	405–415 410	512–515 513.5	19.5–20.7 20.1	55–68 61.5	203–287 245	203–208 205.5	188–212 200
S2Mo Wire with Ni-LaF_3_ Coating	418–420 419	580–590 519.5	19.4–20.2 19.8	66–82 74	256–290 273	218–232 225	209–219 214

## Data Availability

Data sharing is not applicable.
